# Spatial and Temporal Variability in Prevalence Rates of Members of the *Borrelia burgdorferi* Species Complex in *Ixodes ricinus* Ticks in Urban, Agricultural and Sylvatic Habitats in Slovakia

**DOI:** 10.3390/microorganisms11071666

**Published:** 2023-06-27

**Authors:** Mária Kazimírová, Lenka Mahríková, Zuzana Hamšíková, Michal Stanko, Maryna Golovchenko, Natalie Rudenko

**Affiliations:** 1 Institute of Zoology, Slovak Academy of Sciences, Dúbravská cesta 9, 84506 Bratislava, Slovakiastankom@saske.sk (M.S.); 2 Institute of Parasitology, Slovak Academy of Sciences, Hlinkova 3, 04001 Košice, Slovakia; 3 Biology Centre Czech Academy of Sciences, Institute of Parasitology, Branišovská 31, 37005 České Budějovice, Czech Republic; marina@paru.cas.cz (M.G.); natasha@paru.cas.cz (N.R.)

**Keywords:** *Ixodes ricinus*, *Borrelia burgdorferi*, prevalence, species, Slovakia

## Abstract

Lyme borreliosis (LB) is the most prevalent tick-borne human infection in Europe, with increasing incidence during the latest decades. Abundant populations of *Ixodes ricinus,* the main vector of the causative agent, spirochetes from the *Borrelia burgdorferi* sensu lato (*Bbsl*) complex, have been observed in urban and suburban areas of Europe, in general, and Slovakia, particularly. Understanding the spread of infectious diseases is crucial for implementing effective control measures. Global changes affect contact rates of humans and animals with *Borrelia*-infected ticks and increase the risk of contracting LB. The aim of this study was to investigate spatial and temporal variation in prevalence of *Bbsl* and diversity of its species in questing *I. ricinus* from three sites representing urban/suburban, natural and agricultural habitat types in Slovakia. *Ixodes ricinus* nymphs and adults were collected by dragging the vegetation in green areas of Bratislava town (urban/suburban habitat), in the Small Carpathians Mountains (natural habitat) (south-western Slovakia) and in an agricultural habitat at Rozhanovce in eastern Slovakia. *Borrelia* presence in ticks was detected by PCR and *Bbsl* species were identified by restriction fragment length polymorphism (RFLP). *Borrelia burgdorferi* s.l. species in coinfected ticks were identified by reverse line blot. Significant spatial and temporal variability in prevalence of infected ticks was revealed in the explored habitats. The lowest total prevalence was detected in the urban/suburban habitat, whereas higher prevalence was found in the natural and agricultural habitat. Six *Bbsl* species were detected by RFLP in each habitat type —*B. burgdorferi* sensu stricto (s.s.), *B. afzelii*, *B. garinii*, *B. valaisiana*, *B. lusitaniae* and *B. spielmanii*. Coinfections accounted for 3% of the total infections, whereby *B. kurtenbachii* was identified by RLB and sequencing in mixed infection with *B. burgdorferi* s.s, *B. garinii* and *B. valaisiana.* This finding represents the first record of *B. kurtenbachii* in questing *I. ricinus* in Slovakia and Europe. Variations in the proportion of *Bbsl* species were found between nymphs and adults, between years and between habitat types. Spatial variations in prevalence patterns and proportion of *Bbsl* species were also confirmed between locations within a relatively short distance in the urban habitat. Habitat-related and spatial variations in *Borrelia* prevalence and distribution of *Bbsl* species are probably associated with the local environmental conditions and vertebrate host spectrum. Due to the presence of *Borrelia* species pathogenic to humans, all explored sites can be ranked as areas with high epidemiological risk.

## 1. Introduction

Lyme borreliosis (LB) is a multisystemic disorder with diverse clinical manifestations caused by spirochaetes of the *Borrelia burgdorferi* sensu lato (s.l.) (*Bbsl*) complex. In Europe, LB is the most prevalent tick-borne human infection, transmitted by *Ixodes ricinus,* the main vector of the disease. The *Bbsl* complex currently includes 23 recognised validated and proposed species, the majority of which occur in the temperate zones of the Northern Hemisphere. Nine species are present in Europe, viz., *B. burgdorferi* sensu stricto (s.s.), *B. garinii*, *B. afzelii*, *B. valaisiana*, *B. lusitaniae*, *B. bavariensis*, *B. spielmanii*, *B. turdi* and *B. bissettiae* [1,2,3], while the validity of *B. finlandensis* has recently been questioned [3]. Additional species such as *B. carolinensis*, *B. californiensis*, *B. americana* and *B. lanei* have sporadically been detected in European questing and host feeding *Ixodes* spp. and in wild animal tissues [4,5,6]. In Europe, pathogenicity to humans has been confirmed for eight species (*B. burgdorferi* s.s., *B. garinii*, *B. afzelii*, *B. bavariensis*, *B. spielmanii*, *B. bissettiae*, *B. lusitaniae* and *B. valaisiana*) [7]. Even though associations of a few other species with human cases have been anticipated [3,8], *B. afzelii* and *B. garinii* are responsible for the majority of contribution to human LB in Europe. Both species have been found to be the most prevalent among questing *I. ricinus* ticks in Central Europe and are associated with different clinical manifestations of the disease in humans [1,8,9,10]. 

The ecology of LB in Europe is very complex. Environmental factors affecting the phenology of the vector tick *I. ricinus* and vertebrate species abundance and diversity have been assumed to be the main drivers determining the presence and prevalence of *Bbsl* species in a particular site [3,11,12,13]. However, recently, it has been suggested that only a few widespread vertebrate species (rodents—Rodentia, thrushes—Turdidae, deer—Cervidae) maintain *I. ricinus* and *Bbsl* populations in an environment [14,15]. *Ixodes ricinus* is a generalist tick species with about 300 different vertebrate host species, some of which are competent reservoirs for *Bbsl* [16]. Such a diversity and quantity of hosts may contribute to tick dispersion and lead to intensive colonization of new areas and establishment of new enzootic LB foci. Studies on reservoir competence have indicated associations between *Borrelia* species and their vertebrate hosts [17]. Specific associations were identified, e.g., between small rodents such as mice, voles (Murinae, Arvicolinae) and *B. afzelii* [18]; squirrels (Sciuridae) and *B. burgdorferi* s.s. [19]; birds (Aves) and *B. garinii* and *B. valaisiana* [20,21]; lizards (Lacertidae) and *B. lusitaniae* [22,23]; or between dormice (Gliridae) and *B. spielmanii* [24]. However, the natural circulation and transmission patterns of different *Bbsl* species have still not been fully elucidated. The situation is even more complicated due to existence of multiple diverse strains within the individual *Borrelia* spp. [25]. Expansion of host specialized *Borrelia* spp. (e.g., *B. afzelii* or *B. garinii*) in different geographic regions results in geographic structuring of the pathogen and depends on the host distribution. In contrast, spirochete species without host specialization (e.g., *B. burgdorferi* s.s. or *B. bissettiae*) might infect phylogenetically distant hosts, sometimes covering as many as three orders, generating a spatially uniform population structure [26,27].

*Ixodes ricinus* has a significant impact on public health, being a major vector of multiple human diseases in Europe. Distribution and phenology of *I. ricinus* are influenced by various biotic (host species, host density and behaviour, vegetation structure) and abiotic factors (temperature, relative humidity, altitude, latitude), which are the main drivers determining the spatial and temporal changes in prevalence and genetic variability of *Bbsl* [3,9]. Some biotic and abiotic factors can be effectively managed to decrease the risk of tick bites [28]. *Ixodes ricinus* is an exophilic tick. It actively quests for hosts and its presence is strongly associated with specific biotopes. *Ixodes ricinus* mostly occurs in humid habitats, such as deciduous and mixed forests or forest edges with dense undergrowth and permanent leaf litter [29]. During the last decades, spread and increasing abundance of *I. ricinus* populations have been observed in urban and peri-urban areas of Europe [28,30]. In small urban parks and cemeteries, absence of large mammals serving as feeding hosts for all developmental stages of *I. ricinus* results in changes in tick–host associations as well as in the prevalence and distribution of some pathogens including *Borrelia.* Small- to medium-sized mammals, such as mice, voles, shrews (Soricidae), hedgehogs (Erinaceinae), squirrels, dormice, hares (Lagomorpha), foxes, domestic dogs (Canidae) and cats (Felidae), as well as birds (mostly competent reservoirs of *Borrelia*) become the main hosts for ticks. In contrast, high population densities of roe deer (*Capreolus capreolus*) in large city parks and peri-urban areas determine the establishment and maintenance of abundant tick populations. Deer (Cervidae) and other large mammals that are incompetent hosts for *Borrelia* were found to act as “dilution” hosts, which decrease infection prevalence of *Bbsl* in tick populations; however, their presence can still increase the risk for human populations to acquire LB [15,31].

Similar to the entirety of Europe, increasing incidence of human LB cases and their associations with *B. afzelii* and *B. garinii* have been reported in Slovakia during the last decades [32]. *Borrelia burgdorferi* s.l.-infected questing *I. ricinus* ticks have been found in different habitat types throughout Slovakia, with considerable spatial variations in prevalence and distribution of *Bbsl* species [18,20,21,23,33,34,35,36,37,38,39,40,41,42]. Presence of *B. burgdorferi* s.s., *B. garinii*, *B. afzelii*, *B. valaisiana*, *B. lusitaniae*, *B. bavariensis*, *B. spielmanii* and *B. bissettiae* has been confirmed [18,20,21,23,35,36,37,38,39,40,41,42,43,44]. Associations were found between *B. afzelii*, *B. garinii* and *B. lusitaniae* with small rodents [18], birds [20,21] and lizards [23], respectively, but more investigations are needed to unravel the factors that determine the differences in prevalence and distribution of *Bbsl* species and strains in habitats with various diversities of vertebrate hosts. 

The main objectives of the present study were (i) assessment of prevalence of *Bbsl*-infected questing *I. ricinus* ticks in three habitat types in Slovakia with different anthropogenic impact; (ii) comparison of *Bbsl* prevalence in the three habitat types between three consecutive years; (iii) identification of *Bbsl* species and assessment of their diversity and spatial distribution.

## 2. Materials and Methods

### 2.1. Study Sites and Tick Collections

The research was carried out in the timeframe of the FP7 project EDENext (www.edenext.eu (accessed on 1 February 2011)), with the aim to investigate prevalence and spread of tick-borne diseases in various habitats in Europe. The methods used were standardised in the frame of the project [45]. 

**Figure 1:**
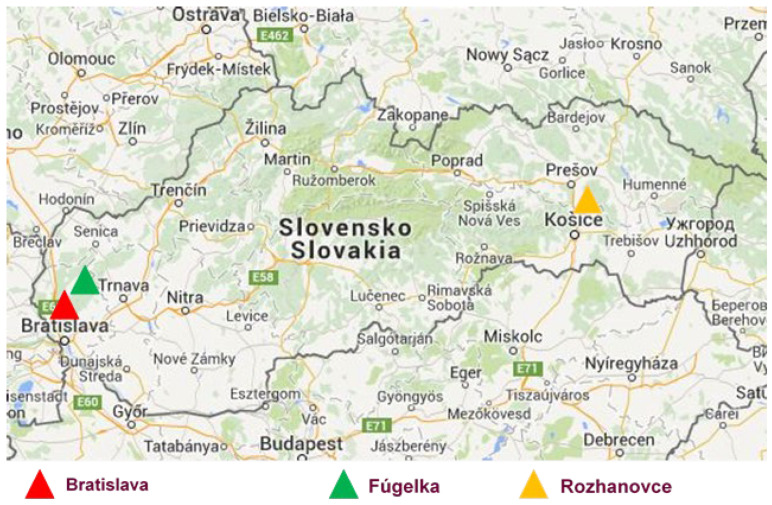
Tick collection sites in Slovakia. Bratislava—urban/suburban habitat, Fúgelka—natural habitat, Rozhanovce—agricultural habitat.

Three study sites comprising different habitat types were selected (Figure 1; [45]). Two sites (urban/suburban and natural habitat) were located on the south-eastern slopes of the Small Carpathians Mountains (south-western Slovakia). The urban/suburban habitat (48.166667 N, 17.066667 E) comprised green patches in the north-western part of Bratislava town, including the campus of the Slovak Academy of Sciences (SAS) and Železná studnička (located in the Bratislava forest park recreational area), which are characterized by moderate-to-significant human intervention and high frequency of visitors. The site representing the natural habitat (48.366667 N, 17.300000 E) was located in a non-fragmented deciduous forest at Fúgelka, about 40 km away from Bratislava. The site was visited predominantly by forest workers, gamekeepers, hunters and hikers (for detailed characterisation of the sites, see [46,47]). The third site was located in eastern Slovakia at Rozhanovce village (Košická kotlina basin; 48.750000 N; 21.366667 E), within a game reserve, with a special facility for breeding mouflon (*Ovis aries musimon*) and fallow deer (*Dama dama*) along with a pheasantry. Rozhanovce is an agricultural habitat with patches of oak–hornbeam forest, i.e., ecotones between the forest and the cultivated fields and meadows (for details, see [48]), and is long-term monitored for diverse pathogen foci [49]. 

Questing ticks were collected by dragging of vegetation with a 1 m^2^ blanket along three 100 m transects set in each habitat. Random additional collections were carried out within close vicinity of the transects. Ticks were collected in monthly intervals during the season of the highest activity of *I. ricinus* (April–June) and in September–October of 2011–2013 (for details, see [46,47,48]). Ticks were stored in 70% ethanol and identified according to species and developmental stage according to Siuda [50] and Filippova [51]. Only *I. ricinus* were selected for molecular analyses. 

In addition to the transects in Bratislava which were sampled regularly during three consecutive years (A, D, E), two transects (B, C) were selected for random tick collections from April to June 2011 (Figure S1). 

The airline distances between the transects are as follows: 350 m between A and B, 420 m between B and C, 2.2 km between C and D and 1.5 km between D and E. The overall airline distance between transects A and E is about 4.5 km. For detailed characterization of the transects, see papers by Svitálková et al. [46] and Mydlová et al. [52]. 

### 2.2. DNA Isolation

Genomic DNA was isolated from individual ticks by using the Macherey-Nagel NucleoSpin^®^ Tissue kit (Düren, Germany), following the manufacturer protocol. Quantity and quality of total DNA were measured with a Nanodrop 2000 c spectrophotometer and samples were stored at −20 °C for further analyses [46]. DNA isolation, subsequent PCR amplification and post-amplification analyses were set up in separate areas with all precautions against contamination.

### 2.3. PCR Amplification and Molecular Analysis

Molecular analyses were performed in all adult ticks and in randomly selected nymphs (whenever possible, in at least 30 individuals per transect and collection date). Total tick DNA was tested for the presence of *B. burgdorferi* s.l. DNA by PCR amplification of a 222–255-bp fragment of the 5S–23S rRNA intergenic spacer [53]. The primers used were IGSA 50-CGACCTTCTTCGCCTTAAAGC-30 and IGSB 50-AGCTCTTATTCGCTGATGGTA-30. The PCR reaction was carried out in a reaction volume of 25 µL in a BioRad T100^TM^ Thermal Cycler (Hercules, CA, USA) by using the HotStarTaq PCR kit (Qiagen, Hilden, Germany). The PCR reaction mixture per sample contained 2.5 μL of Coral Load PCR buffer (containing 15 mM MgCl_2_), 1 μL of MgCl_2_ (25 mM), 0.125 µL of HotStarTaq Plus DNA Polymerase (5 U/µL; Qiagen, Hilden, Germany), 0.5 µL of each primer (10 µM), 0.5 µL of 10 mM dNTP (Thermo Fisher Scientific, Dreieich, Germany) and 14.875 µL of nuclease-free water. Then, 5 µL of total tick DNA was added to the reaction mixture as a template. Negative (nuclease-free water) and positive controls (tick DNA positive for *B. burgdorferi* s.l.) were included in each run. The PCR was set up according to the protocol by Derdáková et al. [53]. The PCR products were electrophoresed on a 1.5% agarose gel stained with GoodView^TM^ Nucleic Acid stain (SBS Genetech, Beijing, China) and visualised with a UV transilluminator. In PCR positive samples, borrelial species were identified by restriction fragment length polymorphism (RFLP) analysis [53]. PCR products were digested with FastDigest^®^ Tru1I restriction enzyme (Fermentas, Thermo Fisher Scientific Biosciences, Leon-Rot, Germany), according to the instructions of the manufacturer. Restriction fragments were analysed by electrophoresis using precast gels (Spreadex gel EL300 mini, Elchrom Scientific, Cham, Switzerland) in the Origins submarine electrophoresis system (Elchrom Scientific, Cham, Switzerland). Gels were stained with SYBRGreen (Sigma-Aldrich, St. Louis, MO, USA) and visualised by UV transilluminator.

### 2.4. Identification of Borrelia Species in Samples with Mixed Infections

The presence of multiple species of LB spirochetes in tick samples was detected by initial PCR amplification. To identify the spirochete species, amplification of the partial genes encoding flagellin and outer surface protein C (*ospC*) was conducted, followed by sequencing of amplicons. To avoid the inhibition of the reaction by the excess of tick DNA, all PCR reactions were conducted in two steps—the first (spacer) and the second (nested) using the protocol previously described [54]. As a template for the second step, 5 µL of the resulting first-step reaction mixture was used. In case of overlap of sequence chromatograms that confirmed the presence of multiple infections in a tick, the cloning step of total PCR product into the TOPO TA cloning vector (Invitrogen, Thermo Fisher Scientific, Waltham, MA, USA) was conducted as described below. 

The fragment of *ospC* gene was amplified by spacer/nested PCR using the protocol of Bunikis et al. [55]. The first round of PCR was conducted using the primers F1, 5′-ATGAAAAAGAATACATTAAGTGC-3′ (positions 306–328 of U01894) and R1, 5′-ATTAATCTTATAATATTGATTTTAATTAAGG-3′ (963–933), targeting 657 bp-long amplicon. The second round of PCR was conducted using the primers F2, 5′-TATTAATGACTTTATTTTTATTTATATCT-3′ (331–359) and R2, 5′-TTGATTTTAATTAAGGTTTTTTTGG-3′ (948–924), targeting 617 bp-long fragment. The conditions of both reactions were the same: 30 cycles at 95 °C for 30 s, 52 °C for 30 s and 72 °C for 30 s, except for the annealing temperature, which was 50 and 52 °C for the first and the second rounds of PCR, respectively.

Primers described by Clark et al. [56] were used to amplify the fragment of *flagellin* gene—F1: 5′- AARGAATTGGCAGTTCAATC-3′ (271–290) and R1: 5′- GCATTTTCWATTTTAGCAAGTGATG-3′ (743–767), targeting 465 bp-long fragment in the first round of PCR, and F2: 5′- ACATATTCAGATGCAGACAGAGGTTCTA -3′ (301–328) and R2: 5′- GAAGGTGCTGTAGCAGGTGCTGGCTGT-3′ (663–689), targeting 388 bp-long fragment in the second round of PCR. The conditions of PCR reaction were exactly the same as in the case with *ospC* gene PCR amplification.

All PCR reactions were carried out in a final volume of 20 μL using 2x HotStarTaq Plus Master Mix (Qiagen, Hilden, Germany). Amplicons were visualized by electrophoresis in a 1% agarose gel (1×  TAE, pH 8.0). SYBR^®^ Gold DNA stain (Invitrogen, Thermo Fisher Scientific, Waltham, MA, USA) was included in the sample loading buffer before the loading of samples into the gel. In all cases, a reaction mixture with water instead of DNA template was used as negative control. Purified DNA from *Bbsl* was used as a positive control in all PCR reactions.

#### 2.4.1. Sequencing

All PCR products of the expected sizes were excised from agarose gels, purified using Ultrafree-DA centrifugal filter units for DNA extraction from agarose (Millipore, Darmstadt, Germany) and sequenced in both directions using the same primers as for PCR. Sequence analysis was performed by SeqMe, (SEQme s.r.o., Dobříš, Czech Republic) and the sequences were compared to those available in the GenBank™ database by Basic Local Alignment Tool (BLAST, https://blast.ncbi.nlm.nih.gov/) (accessed on 1 April 2015) analysis.

#### 2.4.2. Reverse Line Blotting (RLB)

DNA samples were subjected to a conventional PCR, targeting a fragment of the 5S–23S rRNA intergenic spacer (IGS) by use of biotin-linked forward B5S-Bor (biotin-5′-GAGTTCGCGGGAGAGTAGGTTATT-3′), 23SBor reverse (5′-TCAGGGTACTTAGATG GTTCACTT-3′) and biotin-linked forward BMiya-For (biotin-5′-TTAGGATTAATGATRT TKTTACC-3′) primers, as described by Alekseev et al. [57] and Blazejak et al. [58]. PCR cycling conditions included 45 cycles at 94 °C for 20 s, 52 °C for 30 s, 72 °C for 30 s and a final elongation step at 72 °C for 7 min. Each run included different *Borrelia* strains corresponding to the used probes as positive controls (Supplementary file): *B. burgdorferi* s.l. (-), *B. burgdorferi* s.s. (PAbe), *B. garinii* (PWudII), *B. afzelii* (PBas), *B. valaisiana* (VS116), *B. lusitaniae* (Poti B2), *B. spielmanii* (PHap), *B. bissettiae* (DN127), RF-like *Borrelia* (-) and *B. miyamotoi* (HT31). PCR products, as well as positive controls and a negative control, were hybridized to different oligonucleotide probes linked to the membrane (for details, see Supplementary file and [58,59,60]).

#### 2.4.3. Cloning and Transformation of the Competent Cells

For separation of multiple species present as coinfection in one sample, total PCR products were cut from the gel, purified and cloned into pCR-4 TOPO plasmid, followed by the transformation of the competent cells and plating of them on LB/agar medium. TOPO™ TA Cloning™ Kit for Sequencing (Invitrogen, Thermo Fisher Scientific, Waltham, MA, USA) was used. The detailed protocol is given in the Supplementary file. Next, 12 colonies were picked individually from the plates and grown overnight in 10 mL LB/amp liquid medium. The overnight culture was centrifuged and the collected cells were used for plasmid DNA isolation using the QIApred Miniprep Kit for plasmid DNA purification (Qiagen, Hilden, Germany). The plasmid DNAs were checked for the presence of the insert required using the PCR with the specific primers. The plasmids with the confirmed insert were sent for sequencing. 

#### 2.4.4. RFLP Analysis

RFLP analysis of *Borrelia* sequences was performed in silico using the free software available at http://insilico.ehu.es (accessed on 15 June 2015). The partial sequence of *fla* gene was digested with HapII, HhaI, HincII, CelII and DdeI. All RFLP patterns obtained were compared with patterns already published [61,62,63].

### 2.5. Statistical Analysis

Differences in the prevalence of *Bbsl*-infected ticks between habitats, years, developmental stages and sexes, as well as differences in the proportion of *Borrelia* species between habitats, were analysed by Chi-square test or Fisher’s exact test using the PAST programme [64]. Additionally, 95% confidence intervals (95% CI) were calculated individually for each proportion by using the online calculator at the website http://epitools.ausvet.com.au (accessed on 15 February 2023). Association between percentage proportion of nymphs in the local tick populations in Bratislava (transects A–E) and total *Bbsl* prevalence was analysed by Spearman rank correlation using the PAST programme. *p* values < 0.05 were considered significant in all tests. 

## 3. Results

### 3.1. Prevalence of Borrelia burgdorferi s.l. in Questing Ixodes ricinus

**Figure 2:**
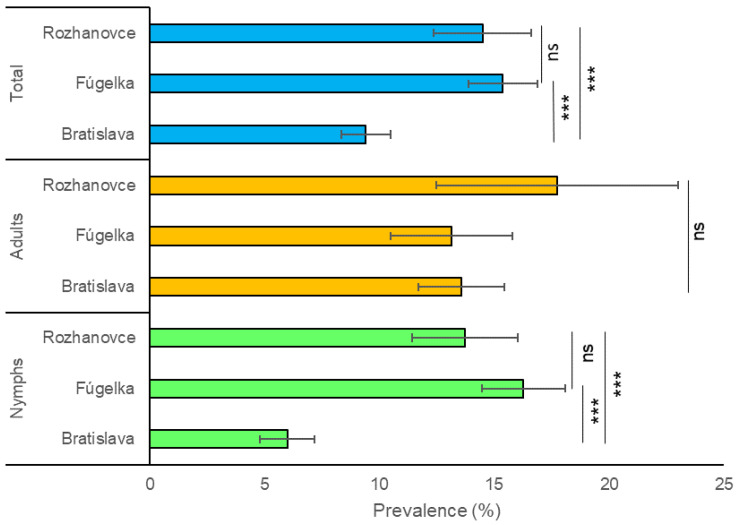
Prevalence of *Borrelia burgdorferi* s.l. in questing *Ixodes ricinus* nymphs and adults per habitat type. Bars represent prevalence with 95% confidence intervals. Significance level of the χ^2^ test: *** *p* < 0.0001, ns—non-significant.

In total, 9071 *I. ricinus* ticks (6954 nymphs, 976 females, 1141 males) were collected during 2011–2013. Of these, 4587 ticks (3305 nymphs, 590 females, 692 males) were collected in Bratislava, 2939 (2307 nymphs, 286 females, 346 males) in Fúgelka and 1545 (1342 nymphs, 100 females, 103 males) in Rozhanovce. A total of 6123 ticks (4006 nymphs, 976 females and 1141 males; 2816 ticks from Bratislava, 2251 from Fúgelka and 1056 from Rozhanovce) were examined, resulting in an overall Bbsl infection prevalence of 12.5% (765/6123; 95% CI: 11.7–13.3%). The total prevalence differed significantly among the three habitats (Table 1; Figure 2): Bratislava 9.4%; Fúgelka 15.4%; Rozhanovce 14.5% (χ^2^ = 44.813; *p* < 0.0001). Prevalence in Bratislava (urban/suburban habitat) was significantly lower than in Fúgelka (natural habitat) and Rozhanovce (agricultural habitat) (χ^2^ = 41.355; *p* < 0.0001 and χ^2^ = 20.236; *p* < 0.0001, respectively), while it did not differ between Fúgelka and Rozhanovce (χ^2^ = 0.437; *p* = 0.509). Similar to overall prevalence, statistical analysis revealed significant differences in the prevalence of *Borrelia*-infected nymphs among the three habitats (χ^2^ = 83.474; *p* < 0.0001) (Figure 2), between Bratislava and Fúgelka (χ^2^ = 82.781; *p* < 0.0001) and Bratislava and Rozhanovce (χ^2^ = 40.882; *p* < 0.0001). The difference between Fúgelka and Rozhanovce was not significant (χ^2^ = 2.745; *p* = 0.097). For adult ticks, infection prevalence did not differ among the habitats (χ^2^ = 2.923; *p* = 0.232) (Figure 2). Significant differences among habitats were also found for infection prevalence in nymphs in individual years (2011: χ^2^ = 46.474; *p* < 0.0001; 2012: χ^2^ = 8.022; *p* = 0.018; 2013: χ^2^ = 31.628; *p* < 0.0001), but not for adult ticks. 

**Figure 3:**
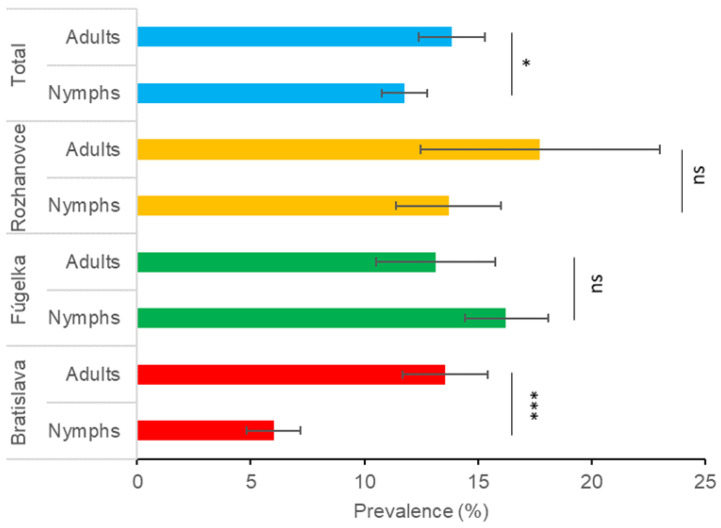
Differences in prevalence of *Borrelia burgdorferi* s.l. between questing *Ixodes ricinus* nymphs and adults in three habitat types. Bars represent prevalence with 95% confidence intervals. Significance level of the χ^2^ test: * *p* < 0.05, *** *p* < 0.0001, ns—non-significant.

The total prevalence of infection in adult ticks (13.8%) was significantly higher than in nymphs (11.8%) (χ^2^ = 5.366; *p* = 0.020). With respect to habitat type, the difference between adults and nymphs was significant in Bratislava (χ^2^ = 46.850; *p* < 0.0001) but not in Fúgelka (χ^2^ = 3.383; *p* = 0.066) nor Rozhanovce (χ^2^ = 2.136; *p* = 0.144) (Figure 3). 

Significantly higher total prevalence was determined in female *I. ricinus* than in males (χ^2^ = 29.364; *p* < 0.0001). The differences between sexes were also significant in Bratislava (χ^2^ = 17.987; *p* < 0.0001) and Fúgelka (χ^2^ = 13.346; *p* < 0.001), while in Rozhanovce the difference was not significant (χ^2^ = 0.216; *p* = 0.642) (Table 1). 

By comparing the prevalence of infection in *I. ricinus* among the three years (2011–2013) separately in each habitat, significant differences were revealed for nymphs and for nymphs + adult ticks in both Bratislava and Fúgelka, as well as for adult ticks in Rozhanovce (Table 1).

### 3.2. Occurrence of Borrelia burgdorferi s.l. Species

**Figure 4:**
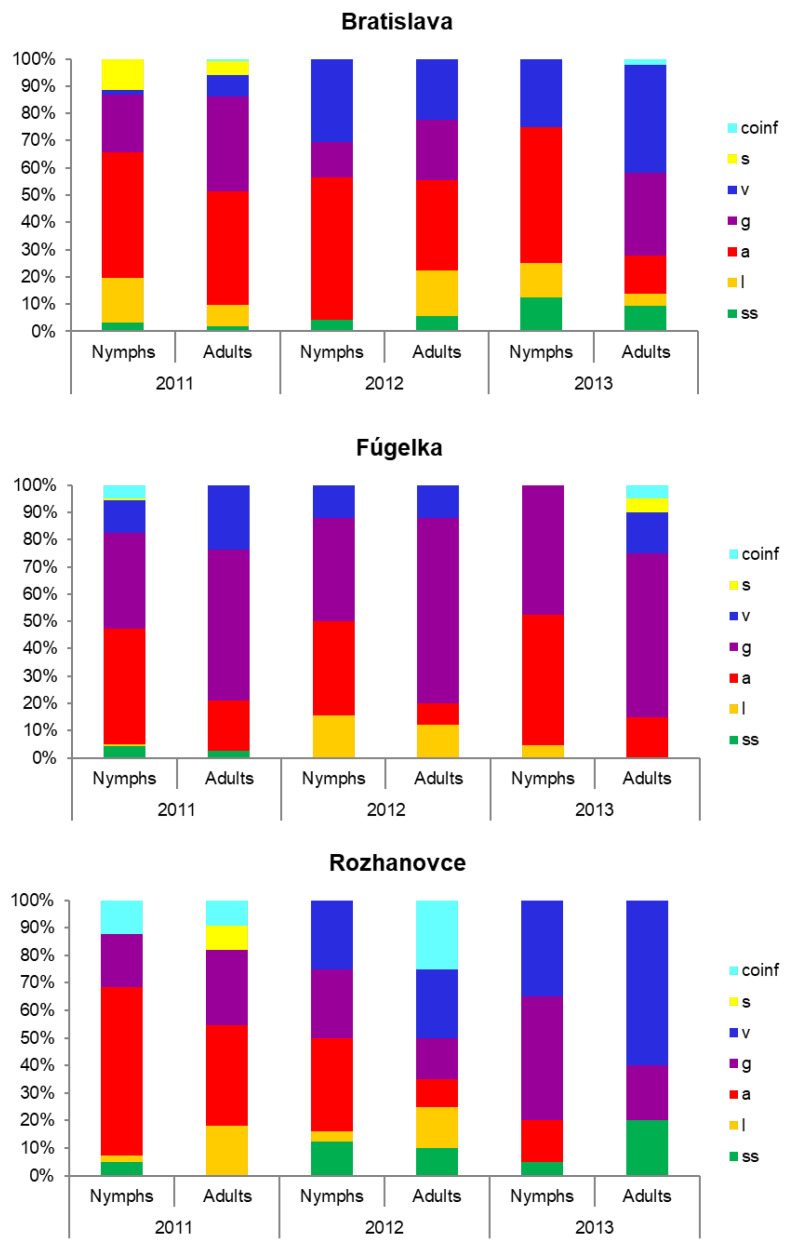
Proportion of *Borrelia burgdorferi* s.l. species (in %) in infected *Ixodes ricinus* nymphs and adults per habitat and year. Species: ss—sensu stricto, l—*lusitaniae*, a—*afzelii*, g—*garinii* (+ *bavariensis*), v—*valaisiana*, s—*spielmanii*, coinf—coinfections.

Overall, six *Bbsl* species were detected by RFLP: *B. burgdorferi* s.s., *B. afzelii*, *B. garinii* (including *B*. *bavariensis*), *B. valaisiana*, *B. lusitaniae* and *B. spielmanii*. Coinfections with two or more species were detected in 23 (3%) out of the 765 *Borrelia*-infected ticks. The presence of the six species was confirmed in each habitat, in nymphs, in adults or both developmental stages and at least in one year (Table 2, Figure 4). *Borrelia afzelii, B. garinii* and *B. valaisiana* were present in all habitats and during all analysed years. Details on the numbers of ticks (nymphs, females, males) infected with the different *Borrelia* species per habitat type and year are presented in Table S1. 

Differences in the proportion of ticks infected with individual *Bbsl* species among habitats were significant for *B. burgdorferi* s.s. (χ^2^ = 15.100; *p* = 0.0005), *B. afzelii* (χ^2^ = 9.051; *p* = 0.011), *B. garinii* (χ^2^ = 53.501; *p* < 0.0001), *B. valaisiana* (χ^2^ = 14.803; *p* < 0.001) and *B. spielmanii* (Fisher’s exact test; *p* = 0.022), while they were not significant for *B. lusitaniae* (χ^2^ = 1.162; *p* = 0.559). Paired comparisons between habitats revealed significant differences in the proportion of *B. burgdorferi* s.s. between Bratislava and Rozhanovce (χ^2^ = 13.167; *p* < 0.001) and between Fúgelka and Rozhanovce (χ^2^ = 7.752; *p* = 0.005), in the proportion of *B. afzelii* between Bratislava and Fúgelka (χ^2^ = 8.343; *p* = 0.004) and between Bratislava and Rozhanovce (χ^2^ = 3.959; *p* = 0.047), in the proportion of *B. garinii* between all habitats (Bratislava vs. Fúgelka χ^2^ = 53.223; *p* < 0.0001; Bratislava vs. Rozhanovce χ^2^ = 8.041; *p* = 0.004; Fúgelka vs. Rozhanovce χ^2^ = 7.911; *p* = 0.005), in the proportion of *B. valaisiana* between Bratislava and Rozhanovce (χ^2^ = 15.031; *p* < 0.001) and between Fúgelka and Rozhanovce (χ^2^ = 4.004; *p* = 0.045) and in the proportion of *B. spielmanii* between Bratislava and Fúgelka (Fisher’s exact test; *p* = 0.017). The proportion of ticks with coinfections differed significantly among the three habitats (Fisher’s exact test; *p* = 0.0001), whereas paired comparisons revealed significant differences between Bratislava and Fúgelka (Fisher’s exact test; *p* = 0.008) and between Bratislava and Rozhanovce (Fisher’s exact test; *p* < 0.0001).

### 3.3. Variability in Infection Prevalence and Distribution of Borrelia burgdorferi s.l. Species in Bratislava

The percentage share of nymphs in the total number of collected ticks varied in the studied transects between 6.7% (C) and 81.2% (A). Out of the 1694 examined ticks from this locality, the presence of *Bbsl* was confirmed in 192 individuals (11.3%; 95% CI: 9.8–12.8%). Significant differences among the transects were found for prevalence of *Borrelia*-infected nymphs and adults as well as for total prevalence (Table 3). A significantly negative association between the percentage proportion of nymphs in the tick populations in transects A–E and total *Borrelia* prevalence was observed (r_s_ = −0.9, *p* = 0.037).

The proportion of the identified *Borrelia* species varied considerably between transects, even between those within a relatively short distance (Figure S2). Four species, *B. afzelii*, *B. garinii*, *B. valaisiana* and *B. lusitaniae*, were found in each of the five transects in nymphs, adults or both. *Borrelia burgdorferi* s.s. was present only in nymphs collected in transect B and *B. spielmanii* in nymphs in transect B, as well as adults in transects A, B and D. Prevalence of *B. afzelii* was the highest (37.5–67.4%) in all transects, except for transect E, where *B. lusitaniae* prevailed (50%)*. Borrelia garinii* followed *B. afzelii* in transects A, C and D (31.2%, 25.6% and 42.9%, respectively). The highest proportion of *B. valaisiana* (18.5%) was determined in transect A, where, together with *B. garinii,* they accounted for 49.5% of infected ticks. Coinfections were detected in two ticks (*B. garinii* + *B. valaisiana* in a female from transect D, not identified in a female from transect C).

### 3.4. Borrelia burgdorferi s.l. Species in Mixed Infections

The presence of multiple spirochete species in ticks that feed few times during their lifespan, mostly on different host species, is not surprising. Identification of species in coinfected samples was performed by RLB, followed by a cloning step and sequencing. In general, by using these combined methods to separate multiple species detected in a single sample, the presence of nine spirochete species was confirmed: *B. burgdorferi* s.s., *B. garinii*, *B. afzelii*, *B. valaisiana*, *B. bissettiae*, *B. spielmanii*, *B. lusitaniae*, *B. kurtenbachii* and *B. bavariensis.* The majority of coinfections (52.2%) in the present study represented samples with paired host associated species, such as bird-associated *B. garinii + B. valaisiana* that occurred in each habitat type. Samples where bird-associated species were accompanied with rodent-associated *B. afzelii* were detected as well, though in a lower ratio (8.7%) (for detailed data, see Table S1). Considering the proportion of *B. garinii + B. valaisiana*, the difference among the three habitats was significant (Fisher’s exact test; *p* = 0.003), whereas paired comparisons revealed a significant difference only between Bratislava and Rozhanovce (Fisher’s exact test; *p* = 0.002). In 30.4% of tick DNA samples for which the RFLP profile indicated mixed infections, only *B. garinii* could be identified by RLB and sequencing, and no DNA was available for further analyses. It is worth mentioning that, in one nymph from Fúgelka (natural habitat), the presence of *B. garinii* + *B. burgdorferi* s.s. was detected and, in another nymph, three species (*B. garinii*, *B. valaisiana*, *B. afzelii*) were identified. In one single female from Rozhanovce (agricultural habitat), the presence of four species of LB spirochetes was detected: *B. burgdorferi* s.s., *B. garinii*, *B. valaisiana* and *B. kurtenbachii.* The presence of *B. kurtenbachii* was confirmed by the results of RLB and the sequence, which was identical to the type strain 25015. To the best of our knowledge, this is the first record of *B. kurtenbachii* in a natural *I. ricinus* population from Slovakia and Europe. 

## 4. Discussion

Spatial and temporal variations in prevalence and genetic diversity of *Bbsl* are phenomena reported from a number of sites across Europe which differ in geographic position, habitat structure, biodiversity of vertebrate species and land-use patterns [65,66,67,68,69,70,71,72,73,74,75,76]. Due to the current distribution, diversity and invasiveness of *Borrelia* spp. in Europe [3,10] and an increasing overlap of human and spirochaete habitats [2,6,30], the risk to contract LB is higher than has been reported. Climate change, affecting abundance and distribution of ticks and vertebrates, prevalence of *Borrelia* infection in ticks, land use patterns and human activities, may have unexpectedly significant impact on risk prediction of LB [26].

In the present study, we investigated the prevalence of infection and distribution of *Bbsl* species in questing *I. ricinus* ticks in three habitat types in Slovakia during three consecutive years. We hypothesised that the proportion of infected ticks and diversity of *Bbsl* species differ between urban/suburban green areas, un-fragmented deciduous forests and agricultural areas due to variations in the structure of the habitats and differences in the diversity of vertebrate species serving as tick feeding hosts and reservoirs for the pathogens. Our results are largely consistent with previous observations. The overall prevalence of *Bbsl* in our study was lower or in agreement with the 13.7% and 12.3% mean prevalence reported in Europe-wide meta-analyses based on prevalence data in questing ticks from 1984–2003 [77] and 2010–2016 [10], respectively, but it was considerably lower than the mean prevalence (~20%) calculated for Central Europe [10,77]. The mean prevalence of infected nymphs in our study was higher or the same as the values calculated in the cited meta-analyses (10.1% and 11.8%, respectively), whereas, for adults, the prevalence was lower than the values 18.6% and 14.9% obtained by Rauter and Hartung [77] and Strnad et al. [10], respectively. 

Generally, *Bbsl* prevalence in nymphs was found to be lower than in adults [10,77], which has been attributed to a higher probability to acquire the pathogen for adults than for nymphs due to transstadial transmission and an additional blood-meal from reservoir hosts. Results of our study follow this trend in the urban and agricultural habitat but, in the natural habitat, prevalence in nymphs was higher than in adults. This could be explained, in part, by the fact that, in the natural habitat, the diversity of feeding hosts which are also competent reservoirs is higher for larvae than in the other habitat types, while nymphs may feed more frequently on species (e.g., deer) that are not competent reservoirs for *Bbsl.* On the other hand, prevalence in adult ticks from the natural and urban habitats was almost the same, which is probably due to the presence of roe deer in some urban locations and connectivity of the other locations with areas where roe deer or other cervids are present. In the agricultural site where ticks were collected inside the enclosure with game (fallow deer, mouflon, pheasant *Phasianus colchicus*) and in the ecotone between fields and oak–hornbeam forest, the *Bbsl* prevalence in adult ticks was higher than in the other explored habitat types, probably due to presence of abundant populations of competent hosts such as rodents and ground-feeding birds [49]. In previous studies, circulation of *Bbsl* in Rozhanovce was confirmed based on presence of antibodies against *Borrelia* in 10.7% of mouflons, 45.8% of fallow deer [78] and in 9–40% of small mammals [49,79]. 

In a study by Rosá et al. [45], aimed at modelling density of questing *I. ricinus* nymphs (DON) and their infection rate with *Bbsl*, *Anaplasma phagocytophilum* and *Rickettsia* spp. in urban, agricultural and natural habitats of Italy, Germany, Czech Republic, Hungary and Slovakia by considering various climatic and environmental factors, the same dataset of nymphs (excluding randomly sampled ticks) from three 100 m-long transects per habitat and period from April to June of 2011–2013 was used, as that analysed in the present study. Interestingly, the highest DON was found in Slovakia (average 518.7 nymphs/year/300 m^2^). By summarising data from all countries, the highest DON was found in the natural habitat, followed by the urban and agricultural habitats, while in Slovakia, the highest DON was found in the urban habitat. The overall prevalence of *Bbsl* in nymphs based on data from Slovakia, Germany and Italy was 19.3% (i.e., a higher value than in the present study). The mentioned findings also suggest that, in the explored urban/suburban habitat of Slovakia, due to presence of tick feeding hosts that are not competent reservoirs of *Borrelia* (e.g., deer), the DON is high but the prevalence of *Bbsl*-infected nymphs is relatively low. 

The proportion of *Bbsl*-infected ticks in previous studies from Slovakia was found to vary, depending on the location and habitat type, with values ranging from 1.0% to 27.8% in nymphs and from 0% to 49.4% in adults, and with a total prevalence ranging between 0% and 38.3% in urban/suburban sites, between 8.0% and 29.4% in natural sites and between 32.3% and 38.1% in agricultural sites and ecotones (Table S2, [80,81,82,83,84]). Thus, our prevalence values for the three studied habitat types are in the lower range limit of the prevalences reported from Slovakia. 

Although the abovementioned meta-analyses [10,77] did not show any significant effect of different PCR-based detection methods on prevalence rates, we came to a different conclusion. In a previous study aimed at determination of mixed infections of *Bbsl* and *B. miyamotoi*, 136 randomly selected ticks from the same sample set as that analysed in the current study were screened for presence of *Bbsl* by qPCR targeting fragments of the *ospA* and *flaB* genes, and species were identified by sequencing [44]. A 21.3% prevalence of infection was determined and, in 13.2% of the samples, *Bbsl* species were identified, while, in the present study, the prevalence rate in the same selected ticks was 9.5%, but species could be identified in all positive samples.

*Borrelia afzelii* and *B. garinii* are the most frequent species in Europe, followed by *B. burgdorferi* s.s., *B. valaisiana* and *B. lusitaniae*; however, their representation was found to vary between regions and countries [9,10,77]. For Central Europe, the mean percentual proportions were as follows: *B. afzelii* (44%), *B. garinii* (23%), *B. burgdorferi* s.s. (15%), *B. valaisiana* (11%), *B. lusitaniae* (6%), *B. spielmanii* (2%), *B. bissettiae* (0.1%) and *B. bavariensis* (2%) [10]. The listed eight species were also reported from Slovakia, with *B. afzelii* prevailing in the majority of the explored sites (Table S2). Considering total prevalence of *Bbsl* species, our results partly agree with previous findings. *Borrelia afzelii* and *B. garinii* were most prevalent, while *B. valaisiana* and *B. lusitaniae* were more prevalent than *B. burgdorferi* s.s. and the proportion of *B. spielmanii* was 2.1%. By the PCR and RFLP method used in our study, identification of *B. bavariensis* was not possible (see above); thus, infections with *B. garinii* may include *B. bavariensis*. The assumption that *B. bavariensis* circulates in the three studied habitats is supported by its previous detection in a yellow-necked mouse (*Apodemus flavicollis*) specimen from Bratislava forest park and in a questing *I. ricinus* female from Fúgelka [44], as well as in *I. ricinus* nymphs (formerly *B. garinii ospA* serotype 4) from Rozhanovce [37]. 

Established foci of *Bbsl* spirochetes should possess an appropriate environment for vector survival, an adequate density of reservoirs and a temporal and spatial overlap between them. The same six *Bbsl* species as mentioned above were detected in the three studied habitats, suggesting that, in all of them, suitable conditions for their circulation exist. In the present study, the proportion patterns of the *Bbsl* species varied according to habitat and tick developmental stage, as well as between years. The proportion of *B. afzelii* was mostly higher in nymphs than in adult ticks, probably because larvae fed preferably on rodents while nymphs fed on other hosts. In the natural habitat, bird-associated species (*B. garinii*, *B. valaisiana*) prevailed. This may suggest that a higher portion of ticks fed on birds than in the urban/suburban habitat, although rodents were abundant there in 2012, while they declined in 2013 after a long and cold winter [46]. The marked temporal changes in the *Bbsl* species distribution patterns, with a decline in the proportion of *B. afzelii* in the agricultural habitat, are difficult to explain. Fluctuations in rodent populations, phenology of *I. ricinus* and presence of a pheasantry could partly account for the changing patterns in favour of bird-associated species. 

Coinfections in tick vectors and reservoir hosts with multiple *Borrelia* species are common in Europe. The most prevalent (51%) are coinfections with the bird-associated species *B. garinii* + *B. valaisiana,* followed by *B. afzelii* and *B. burgdorferi* s.s. [77]; however, triple and quadruple infections with species associated with different hosts have also been detected in single ticks (e.g., [68,70,85]). Coinfections with *B. garinii* and *B. valaisiana* were found to prevail also in Slovakia, evidenced in [20,21,42] as well as in the present study. Recently, in neighbouring countries to Slovakia, the presence of “atypical” spirochete species, or their combinations, were detected in questing ticks in Poland [4] or in vertebrate hosts in the Czech Republic [6]. For the first time, the coinfection with *B. bissettiae* and *B. carolinensis* was shown in cadavers of red squirrels (*Sciurus vulgaris*) and European hedgehogs (*Erinaceus europaeus*). Using the multiple analyses of several tissues from cadavers, mixed infections of two or three species were shown rather often in northern white-breasted hedgehogs (*Erinaceus roumanicus*, including *B. burgdorferi* s.s. + *B. afzelii* + *B. garinii* in one animal) [6]. Previously considered as “rodent associated”, *B. bavariensis* and *B. afzelii,* were detected in tissues of common blackbirds (*Turdus merula*), together with the expected *B. garinii, B. valaisiana* and *B. turdi* [6]. In a single questing *I. ricinus* nymph from Slovakia, mixed infection with *B. afzelii* + *B. garinii* + *B. bissettii* was confirmed [20]. Detection of unexpected *B. kurtenbachii* as one of the species in a coinfected sample in the present study may be in line with our speculation about the role of migrating hosts in distribution of the causative agents of LB. Another explanation may be that *B. kurtenbachii*, formerly known as *B. bissettii* strain 25015 from human samples from Europe [86,87], has circulated in natural *I. ricinus* populations for a long time, but has remained unrecognized due to its low prevalence and insufficient detection methods. 

Due to increasing exposure of humans and domestic animals to tick bites in urban parks, gardens, cemeteries and peri-urban recreational areas, especially during the last decades, these habitats are of special interest from an epidemiological point of view [30,88]. In a number of studies, the presence and prevalence of *B. burgdorferi* s.l. in *I. ricinus* ticks from urban and peri-urban areas in Europe have been reported. The findings from the period 1990–2021 were reviewed by Hansford et al. [88], who calculated a 17.3% mean prevalence of *Borrelia* infection—14.2% in nymphs and 21.1% in adult ticks. In our study, the total prevalence, as well as the prevalence in nymphs and adults in the urban/sub-urban habitat, were lower than the European average. Total prevalence in our study was also in the lower limit of previously published values from green areas of Bratislava and other urban areas in Slovakia [38,40,41] (Table S2). 

Marked spatial variations in total prevalence of infected ticks have been reported from a number of European cities. For example, in Budapest, prevalence ranged from 31.3% in a cemetery to 50% in an urban forest [89] and values ranging from 1.8% to 60.5% were determined in 11 green urban locations in Prague [90]. In 10 different recreational areas in Hanover, *Borrelia* infection rates in *I. ricinus* varied between 14.8% and 40.5% [58,91]. In two green areas in Munich, 20.1% and 30.1% of ticks were infected [70], while, in another study, seven green plots were explored and the *Bbsl* prevalence varied from 17.4 to 59.5% in nymphs and from 0 to 61.5% in adults [92]. In two cities in Poland, Olsztyn and Warsaw, the proportion of infected ticks ranged between 18.2 and 25% [71] and 7.7 and 14% [93], respectively. Great spatial variability in the spectrum of *Bbsl* species and in their proportions, mostly with predominance of *B. afzelii* or *B. garinii*, were also reported for the mentioned cities. 

Spatial variability in *Borrelia* prevalence has been reported also in Slovak cities. For example, 1.8–18% and 0–33.3% total prevalences were determined in different green locations of Košice and Bardejov (eastern Slovakia), respectively [38], while, in green areas of Bratislava, prevalence of infected ticks varied between 6.8 and 30.9% [40,41]. Thus, spatial differences in *Borrelia* prevalence and distribution of *Bbsl* species within the same cities are common and are likely affected by the local environmental conditions, microclimate, structure of the biotope, biodiversity of vertebrate hosts, connectivity of the locations to forested areas, etc. However, in some cases, the results may be biased due to low numbers of examined ticks. In general, our results on the spatial variability in *Bbsl* prevalence between the five locations (A–E) in Bratislava (see Figure S2) are in line with previous findings on the mosaic patterns of *Bbsl* infection rates in ticks from different patches of the same area [94,95]. The situation in the SAS campus where two sampling transects (A, B) were located at a distance of about 350 m deserves particular attention. The campus is a fenced area with low abundance of small rodents and a local roe deer population that obviously supports the high abundance of *I. ricinus* [46], which may act as a dilution factor for *Borrelia*. Significantly lower *Bbls* prevalence was found in transect A than B (see Table 3), which may suggest that roe deer concentrate mainly around transect A, while they avoid frequent encounters with people in the part of the campus where transect B is located. A third transect within the SAS campus with similar environmental conditions as transect A was sampled in the same period (2011–2012) by Chvostáč et al. [40], who determined a 6.8% prevalence by the same PCR method as applied in our study. The three locations in the SAS campus differed also in the proportion of *Bbsl* species. In the transect studied by Chvostáč et al. [40], bird-associated species predominated, similarly as in transect A, while, in transect B, prevalence of *B. afzelii* was higher than of *B. garinii* + *B. valaisiana*. Thus, even at low abundance of rodents, more than 30% of ticks carried *B. afzelii.* Overall, the findings in the SAS campus indicate that birds, rodents and other small- and medium-sized mammals (e.g., hedgehogs) are the important reservoir hosts for *Borrelia*, while roe deer maintain an abundant *I. ricinus* population. In contrast to the SAS campus, rodents were more abundant in transects D and E, located in Bratislava forest park [46], which was displayed in the high proportion of nymphs infected with *B. afzelii* in transect D. In contrast to transect D, considerably lower prevalence was determined in ticks from transect E (Table 3) (distance between the transects was 1.5 km). Interestingly, 50% of both nymphs and adult ticks collected in transect E were infected with *B. lusitaniae,* suggesting the presence of an abundant lizard population. In comparison with our results, in another location in the forest park at a distance of about 2 km from transect D, a total prevalence of 12.6% with predominance of *B. afzelii* (comparable with transect D) was found [41]. 

## 5. Conclusions

Presence of questing *I. ricinus* ticks infected with *Bbsl* was confirmed during a three-year study in three habitat types in Slovakia. Prevalence of infected ticks was found to differ between the habitats, locations, years and developmental stages (nymphs and adults). Presence of the same six *Borrelia* species was identified in each of the habitats with variable proportions, depending on the habitat type, year and tick developmental stage. The study confirmed that urban/suburban sites and parks can be focal points for circulation of LB spirochaetes. Due to the presence of human-pathogenic *Borrelia* species such as *B. garinii*, *B. afzelii*, *B. burgdorferi* s.s. and *B. spielmanii*, the explored habitats can be ranked among areas with high epidemiological risk. More research is needed to explain circulation patterns of individual *Borrelia* species in habitats with different diversity of reservoirs, as well as on the effect of migrating vertebrate hosts on *Borrelia* prevalence and establishment of new foci of pathogens with impact on public health in highly populated regions. 

## Figures and Tables

**Table 1:** Prevalence of *Borrelia burgdorferi* s.l. in *Ixodes ricinus* per habitat type in 2011–2013.

		2011		2012		2013				Total	
Site		% (pos/ex)	95% CI	% (pos/ex)	95% CI	% (pos/ex)	95% CI	χ^2^	*p*	% (pos/ex)	95% CI
Bratislava	Nymphs	6.9 (61/887)	5.4–8.7	11.6 (23/199)	7.1–16.0	1.8 (8/448)	0.6–3.0	26.227	**<0.0001**	6.0 (92/1534)	4.8–7.2
(urban/suburban habitat)	Females	18.6 (69/370)	14.7–22.6	14.5 (9/62)	5.7–23.3	17.7 (28/158)	11.8–23.7	0.624	0.732	18.0 (106/590)	14.9–21.1
	Males	9.9 (44/443)	7.1–12.7	13.2 (9/68)	5.2–21.3	8.29 (15/181)	4.3–12.3	1.381	0.501	9.8 (68/692)	7.6–12.0
	Adults total	13.9 (113/813)	11.5–16.3	13.8 (18/130)	7.9–19.8	12.3 (43/339)	9.1–16.2	0.310	0.856	13.6 (174/1282)	11.7–15.4
	Total	10.2 (174/1700)	8.8–11.7	12.5 (41/329)	8.9–16.0	6.5 (51/787)	4.8–8.2	12.829	**0.002**	9.4 (266/2816)	8.3–10.5
Fúgelka	Nymphs	17.0 (184/1084)	14.7–19.2	19.5 (58/298)	15.0–24.0	8.9 (21/237)	5.2–12.5	12.190	**0.002**	16.2 (263/1619)	14.4–18.0
(natural habitat)	Females	17.0 (26/153)	11.0–22.9	25.0 (15/60)	14.0–36.0	16.4 (12/73)	7.9–24.9	2.114	0.347	18.5 (53/286)	14.0–23.0
	Males	7.2 (12/167)	3.3–11.1	12.0 (10/83)	5.0–19.0	8.3 (8/96)	2.8–13.9	1.674	0.433	8.7 (30/346)	5.7–11.6
	Adults total	11.9 (38/320)	8.3–15.4	17.5 (25/143)	11.3–23.7	11.8 (20/169)	7.0–16.7	3.065	0.216	13.1 (83/632)	10.5–15.8
	Total	15.8 (222/1404)	13.9–17.7	18.8 (83/441)	15.2–22.5	10.1 (41/406)	7.2–13.0	12.921	**0.002**	15.4 (346/2251)	13.9–16.9
Rozhanovce	Nymphs	15.4 (41/266)	11.1–19.7	12.9 (56/433)	9.8–16.1	13.0 (20/154)	7.7–18.3	0.941	0.625	13.7 (117/853)	11.4–16.0
(agricultural habitat)	Females	21.2 (7/33)	7.3–35.2	27.3 (9/33)	12.1–42.5	8.8 (3/34)	0.0–18.4		0.119 ^F^	19.0 (19/100)	11.3–26.7
	Males	13.3 (4/30)	1.2–25.5	25.6 (11/43)	12.5–38.6	6.7 (2/30)	0.0–15.6		0.101 ^F^	16.5 (17/103)	9.3–23.7
	Adults total	17.5 (11/63)	8.1–26.8	26.3 (20/76)	16.4–36.2	7.8 (5/64)	1.2–14.4	8.158	**0.017**	17.7 (36/203)	12.5–23.0
	Total	15.8 (52/329)	11.9–19.7	14.9 (76/509)	11.8–18.0	11.5 (25/218)	7.2–15.7	2.146	0.342	14.5 (153/1056)	12.4–16.6

(pos/ex)—number of positive/number of examined ticks; 95% CI—confidence interval; *p*—significance level of the χ^2^ test, ^F^ Fisher’s exact test, significant differences are indicated in bold.

**Table 2:** Representation of *Borrelia burgdorferi* s.l. species in *Ixodes ricinus* populations from three habitat types.

Species	ss		a		g		v		l		s		coinf		T
	%	95% CI	%	95% CI	%	95% CI	%	95% CI	%	95% CI	%	95% CI	%	95% CI	
Bratislava															
2011	0.1	0–0.3	4.5	3.5–5.4	3.1	2.3–3.9	0.6	0.2–0.9	1.1	0.6–1.6	0.8	0.3–1.2	0.06	0–0.2	1700
2012	0.6	0–1.45	5.5	3.0–7.9	2.1	0.6–3.7	3.3	1.4–5.39	0.9	0–1.9	0		0		329
2013	0.6	0–1.2	1.3	0.5–2.0	1.6	0.8–2.5	2.4	1.3–3.5	0.4	0–0.8			0.1	0–0.4	787
Total	0.3	0.1–0.5	3.7	3.0–4.4	2.6	2.0–3.2	1.4	1.0–1.9	0.9	0.5–1.2	0.5	0.2–0.7	0.1	0–0.2	2816
Fúgelka															
2011	0.6	0.2–1.1	6.5	4.8–7.3	6.1	4.9–7.4	2.2	1.4–3.0	0.1	0–0.2	0.1	0–0.2	0.6	0.2–1.1	1404
2012	0		5.0	3.0–7.0	8.8	6.2–11.5	2.3	0.9–3.7	2.7	1.2–4.2	0		0		441
2013	0		3.2	1.5–4.9	5.4	3.2–7.6	0.7	0–1.6	0.2	0–0.7	0.2	0–0.7	0.2	0–0.7	406
Total	0.4	0.1–0.7	5.3	4.4–6.3	6.5	5.5–7.5	1.9	1.4–2.5	0.6	0.3–0.9	0.1	0–0.2	0.4	0.2–0.7	2251
Rozhanovce															
2011	0.6	0–1.4	8.8	5.7–11.9	3.3	1.4–5.3			0.9	0–1.9	0.3	0–0.9	1.8	0.4–3.3	329
2012	1.8	0.6–2.9	4.1	2.4–5.8	3.3	1.8–4.9	3.7	2.1–5.4	1.0	0.1–1.8	0		1.0	0.1–1.8	509
2013	0.9	0–2.2	1.4	0–2.9	4.6	1.8–7.4	4.6	1.8–7.4	0		0		0		218
Total	1.2	0.6–1.9	5.2	3.7–6.3	3.6	2.5–4.7	2.7	1.8–3.7	0.8	0.2–1.3	0.1	0–0.3	1.4	0.4–1.6	1056
TOTAL	0.5	0.3–0.7	4.5	4.0–5.0	4.2	3.7–4.7	1.8	1.5–2.2	0.8	0.5–1.0	0.3	0.1–0.4	0.4	0.2–0.5	6123

*Borrelia burgdorferi* s.l. species: ss—sensu stricto, l—*lusitaniae*, a—*afzelii*, g—*garinii* (+ *bavariensis*), v—*valaisiana*, s—*spielmanii*, coinf—coinfections; 95% CI—confidence interval; T—total number of examined ticks.

**Table 3:** Prevalence of *Borrelia burgdorferi* s.l. in *Ixodes ricinus* per transects A–E in Bratislava in 2011.

Transect	Nymphs		Adults		Total		%N
	% (pos/ex)	95% CI	% (pos/ex)	95% CI	% (pos/ex)	95% CI	
A	2.6 (10/381)	1.0–4.2	6.8 (6/88)	1.5–12.1	3.4 (16/469)	1.8–5.0	81.2
B	13.9 (25/180)	8.8–18.9	8.6 (25/291)	5.4–11.8	10.6 (50/471)	7.8–13.4	38.2
C	14.3 (2/14)	0.0–32.6	21.0 (41/195)	15.3–26.7	20.6 (43/209)	15.1–26.0	6.7
D	25.0 (19/76)	15.3–34.7	25.4 (44/173)	18.9–31.9	25.3 (63/249)	19.9–30.7	30.5
E	5.1 (12/233)	2.3–8.0	12.7 (8/63)	4.5–20.9	6.8 (20/296)	3.9–9.6	78.7
χ^2^	58.554		33.947		101.798		
*p*	**<0.0001**		**<0.0001**		**<0.0001**		

(pos/ex)—number of positive/number of examined ticks; 95% CI—confidence interval; %N—percentage share of nymphs in the total number of collected ticks; *p*—significance level of the χ^2^ test, significant differences are indicated in bold.

## Data Availability

All data generated and analysed during this study are included in the published article and its supplementary information files.

## Supplementary Materials

The following supporting information can be downloaded at: https://www.mdpi.com/article/10.3390/microorganisms11071666/s1, Figure S1: Localisation of transects in Bratislava (urban/suburban habitat) for tick collection in 2011; Supplementary file: Probes used for RLB and the *Borrelia* strains used as positive control, RLB experimental procedure, Cloning and transformation of the competent cells; Table S1: Numbers of *Ixodes ricinus* nymphs, females and males infected with species of *Borrelia burgdorferi* s.l. in three habitat types between 2011 and 2013; Figure S2: Proportion of *Borrelia burgdorferi* s.l. species (in %) in infected *Ixodes ricinus* per transects A–E in Bratislava (urban/suburban habitat) in 2011. Species: ss—sensu stricto, l—*lusitaniae*, a—*afzelii*, g—*garinii* (+ *bavariensis*), v—*valaisiana*, s—*spielmanii*, coinf—coinfections; Table S2: Prevalence of *Borrelia burgdorferi* s.l. and diversity of *Borreia* species reported from Slovakia.
