# Supplementary material for: Spatial and Temporal Variability in Prevalence Rates of Members of the Borrelia burgdorferi Species Complex in Ixodes ricinus Ticks in Urban, Agricultural and Sylvatic Habitats in Slovakia
Source: Microorganisms. 2023 Jun 27;11(7):1666. doi: 10.3390/microorganisms11071666 (PMC10383148; doi:10.3390/microorganisms11071666)
Supplement: Supplementary file 1 [file microorganisms-11-01666-s001.zip › Figure S1.pdf]

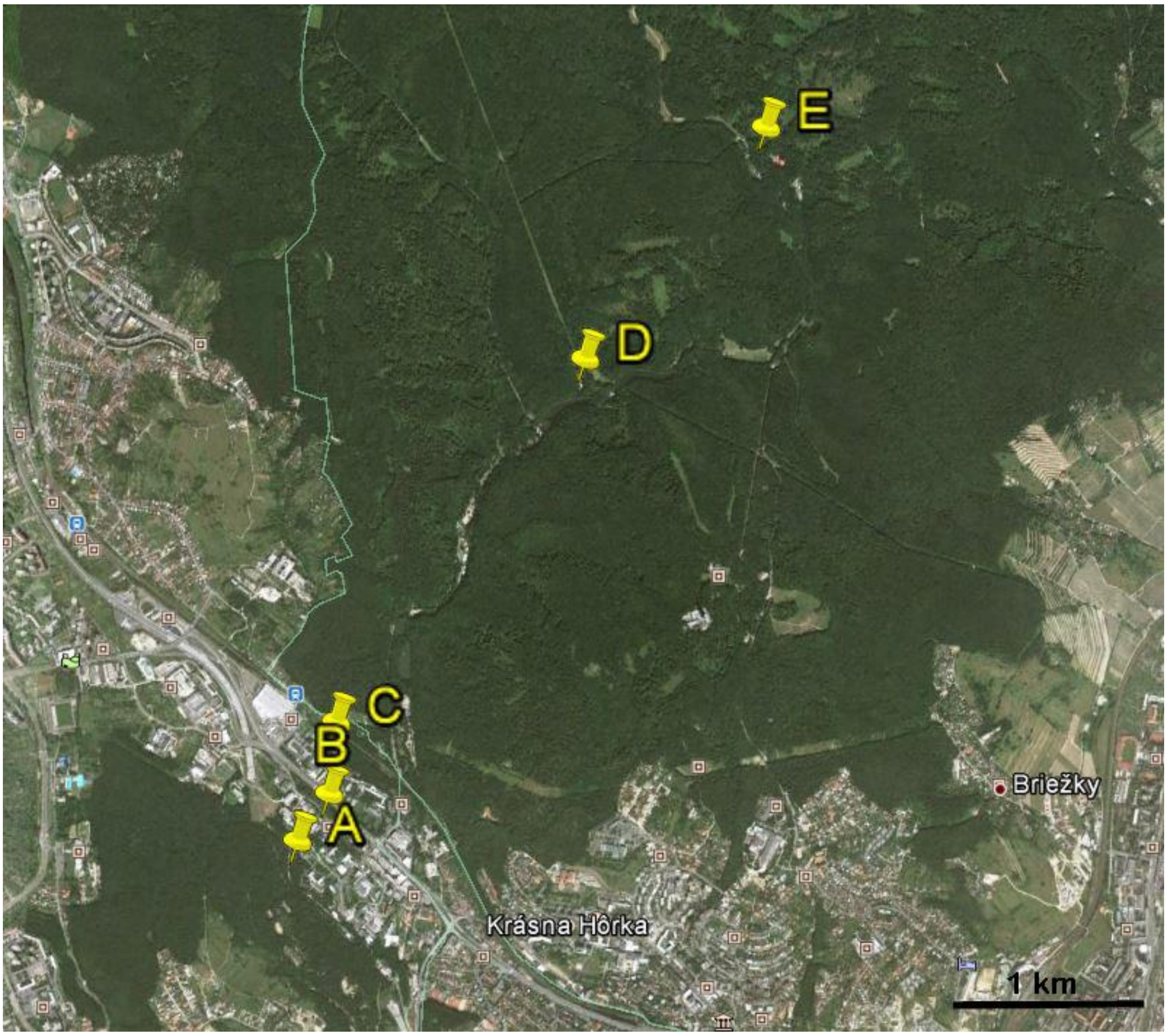


**Figure S1** Localisation of transects in Bratislava (urban/suburban habitat) for tick collection in 2011
