# Supplementary material for: Spatial and Temporal Variability in Prevalence Rates of Members of the Borrelia burgdorferi Species Complex in Ixodes ricinus Ticks in Urban, Agricultural and Sylvatic Habitats in Slovakia
Source: Microorganisms. 2023 Jun 27;11(7):1666. doi: 10.3390/microorganisms11071666 (PMC10383148; doi:10.3390/microorganisms11071666)
Supplement: Supplementary file 1 [file microorganisms-11-01666-s001.zip › Figure S2.pdf]

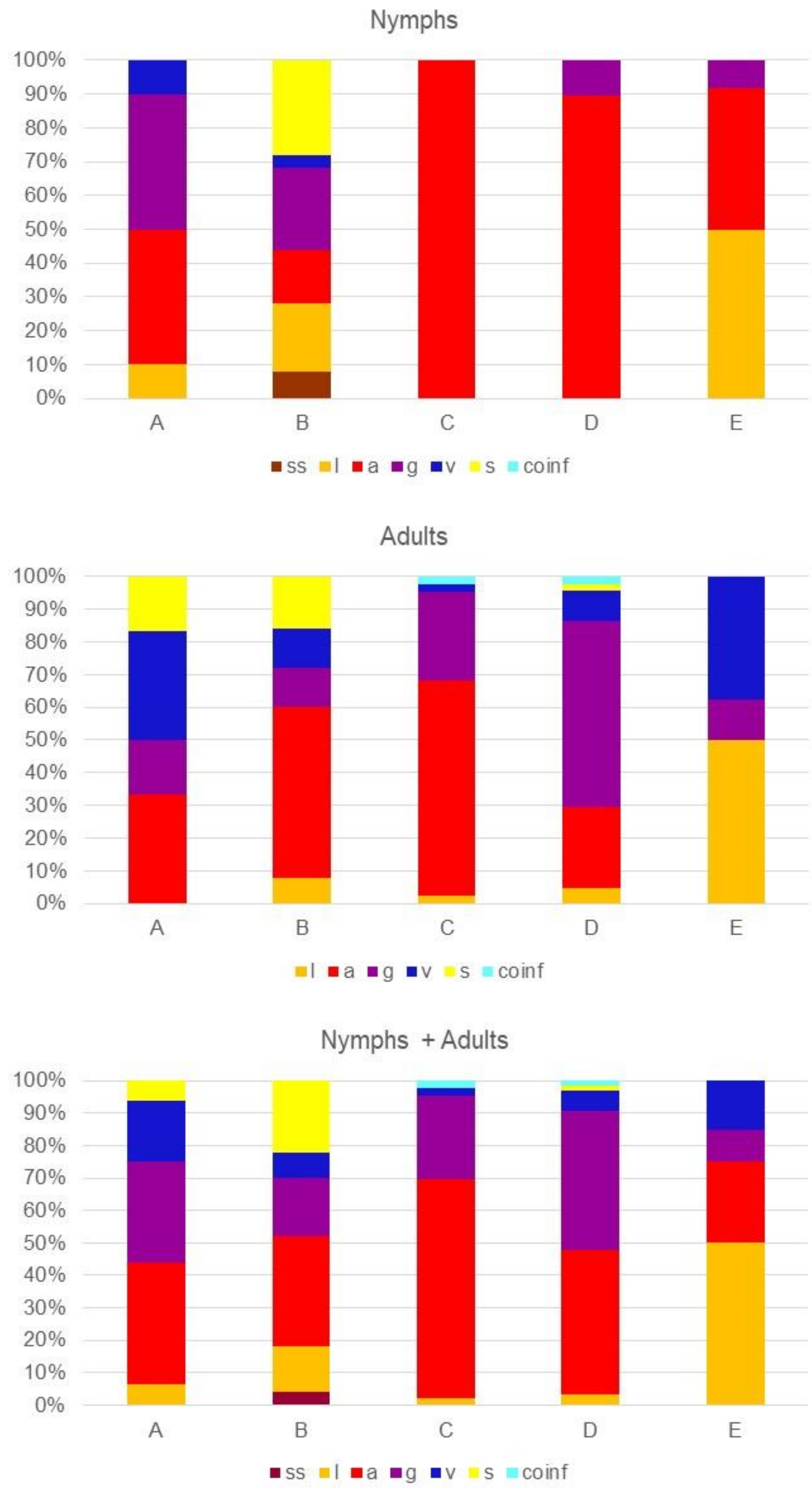

Figure S2 Proportion of *Borrelia burgdorferi* s.l. species (in %) in infected *Ixodes ricinus* per transects A–E in Bratislava (urban/suburban habitat) in 2011. Species: ss – sensu stricto, l – *lusitaniae*, a – *afzelii*, g – *garinii* (+ *bavariensis*), v – *valaisiana*, s – *spielmanii*, coinf – coinfections
