# Supplementary material for: Spatial and Temporal Variability in Prevalence Rates of Members of the Borrelia burgdorferi Species Complex in Ixodes ricinus Ticks in Urban, Agricultural and Sylvatic Habitats in Slovakia
Source: Microorganisms. 2023 Jun 27;11(7):1666. doi: 10.3390/microorganisms11071666 (PMC10383148; doi:10.3390/microorganisms11071666)
Supplement: Supplementary file 1 [file microorganisms-11-01666-s001.zip › Supplementary file.pdf]

**Supplementary file**

**Probes used for RLB and the *Borrelia* strains used as positive control**

| Probe | Reference | Sequence (5' - 3') | Target organism |
|---|---|---|---|
| SL | Gern et al., (2010) | CTTTGACCATATTTTTATCTTCCA | *B. burgdorferi sensu lato* |
| SS | | AACACCAATATTTAAAAAACATAA | *B. burgdorferi sensu stricto* |
| GA | | AACATGAACATCTAAAAACATAAA | *B. garinii* |
| AF | | AACATTTAAAAAATAAATTCAAGG | *B. afzelii* |
| VSNE | | TATATCTTTTGTTCAATCCATGT | *B. valaisiana* |
| LusiNE2 | | AAATCAAACATTCAAAAAAATAAAC | *B. lusitaniae* |
| SpiNE2 | | GAATGGTTTATTCAAATAACATA | *B. spielmanii* |
| SpiNE3 | | GAATAAGCCATTTAAATAACATA | *B. spielmanii* |
| BisNE1 | | AAACACTAACATTTAAAAAACAT | *B. bissettii* |
| BisNE2 | | AACTAACAAACATTTAAAAAACAT | *B. bissettii* |
| RFLNE | | GCATTGCTCAATATGGTTAG | *RF-like* |
| MIYA | Blazejak et al. (2018); Springer et al. (2022) | TGAAAAATTATTTAGTGAAAAGTTC | *B. miyamotoi* |

| Probes | *Borrelia* Strain used as positive control |
|---|---|
| SL | - |
| SS | PAbe |
| GA | PWudII |
| AF | PBas |
| VSNE | VS116 |
| LusiNE2 | Poti B2 |
| SpiNE2 | PHap |
| SpiNE3 | PHap |
| BisNE1 | DN127 |
| BisNE2 | DN127 |
| RFLNE | - |
| MIYA | Pure cloned *B.miyamotoi* DNA (strain HT31) from Russia |

**References**


58. Blazejak, K.; Raulf, M.K.; Janecek, E.; Jordan, D.; Fingerle, V.; Strube, C. Shifts in *Borrelia burgdorferi* (s.l.) geno-species infections in *Ixodes ricinus* over a 10-year surveillance period in the city of Hanover (Germany) and *Borrelia miyamotoi*-specific Reverse Line Blot detection. *Parasit. Vectors* **2018,** *11*, 304. https://doi.org/10.1186/s13071-018-2882-9

59. Gern, L.; Douet, V.; López, Z.; Rais, O.; Cadenas, F.M. Diversity of *Borrelia* genospecies in *Ixodes ricinus* ticks in a Lyme borreliosis endemic area in Switzerland identified by using new probes for reverse line blotting. *Ticks Tick Borne Dis.* **2010**, *1*, 23-29. https://doi.org/10.1016/j.ttbdis.2009.11.001.

60. Springer, A.; Jordan, D.; Glass, A.; Kahl, O.; Fingerle, V.; Girl, P.; Chitimia-Dobler, L.; Strube, C. *Borrelia* infections in ageing ticks: Relationship with morphometric age ratio in field-collected *Ixodes ricinus* nymphs. *Microorganisms*, **2022**, *10*, 166. https://doi.org/10.3390/microorganisms10010166.

**RLB experimental procedure**

1. Activate membrane in 10 ml 16 % 1-ethyl-3-(3-dimethylamino-propyl) carbodiimide (EDAC) (10 min incubation at RT)

2. Wash for 2 min with distilled H2O and place into miniblotter

3. Dilute specific oligonucleotides (probes) (1 µl (100 pmol) of the probe + 349 µl of 500 mM NaHCO3 (pH 8.4)

4. Load oligonucleotides on membrane (also fill empty slots with 500 mM NaHCO3)

5. Incubate for 1 min at RT

6. Aspirate (mark slots with probes with needle)

7. Remove membrane from blotter

8. Inactivate membrane by incubation for 10 min at RT in 100 mM NaOH

9. Rinse membrane with water

10. Block membrane with 125 ml of 2x SSPE-0.1 % sodium dodecyl sulfate (SDS) for 5 min at 60 °C while shaking or block membrane for more than 1 h at 56 °C in 2x SSPE-0.1 % sodium dodecyl sulfate (SDS), 5 % milk while shaking

11. Wash membrane for 5 min at 42 °C with 125 ml 2xSSPE-0.1 % SDS

12. Place membrane into miniblotter with the slots perpendicular on the previously applied specific oligonucleotides (90° rotation of membrane)

13. Add 20 µl of PCR product to 130 µl 2xSSPE-0.1 % SDS, incubate at 100 °C for 10 min

14. Cool on ice immediately

15. Fill the slots

16. Hybridize at 45 °C for 1 h

17. Aspirate PCR products

18. Wash 2x with 2xSSPE-0.5 % SDS for 10 min at 40 °C while shaking

19. Incubate with 1:4000 diluted peroxidase labeled streptavidin in 2x SSPE-0.5 % sodium dodecyl sulfate (SDS) for 30 min at 40 C in the dark (without shaking)

20. Wash 2x for 10 min with 2x SSPE-0.5 % sodium dodecyl sulfate (SDS) at 40 °C while shaking

21. Rinse with 2xSSPE

22. Add TMB stabilized substrate for HRP to detect the positive signals for 10 min in the dark

23. Stop detection with 20 mM EDTA or rinsing the membrane 3x 5 min with H2O

**Cloning and transformation of the competent cells**

<u>Components used for ligation mixture of 6 µl total volume:</u>

| Reagent | Volume [µl] |
|---|---|
| Vector mix | 1 |
| Salt solution | 1 |
| PCR product | 1.5-4 µl ([1]) |
| DNase/RNase-free water | To 6 µl |

[1] Volume dependent on concentration of PCR product [ng/µl]

Mix by inversion and incubate at room temperature for 15-20 minutes

Transformation of competent cells

Add 2 µl of ligation reaction to provided One Shot TOP10 chemically competent *E.coli* cells and mix by inversion

Leave on ice for 20-30 minutes

Incubate at 42°C for 30 seconds

Add 250 µl of SOC medium in sterile conditions

Incubate in shaker at 37°C and 200 rpm for 1 hour. Spread 10-50 µl from transformation reaction on pre-warmed LB/ampicillin plates

Incubate the plates at 37°C overnight without shaking
