# Supplementary material for: Spatial and Temporal Variability in Prevalence Rates of Members of the Borrelia burgdorferi Species Complex in Ixodes ricinus Ticks in Urban, Agricultural and Sylvatic Habitats in Slovakia
Source: Microorganisms. 2023 Jun 27;11(7):1666. doi: 10.3390/microorganisms11071666 (PMC10383148; doi:10.3390/microorganisms11071666)
Supplement: Supplementary file 1 [file microorganisms-11-01666-s001.zip › Table S1.pdf]

**Table S1** Numbers of *Ixodes ricinus* nymphs, females and males infected with *Borrelia burgdorferi* s.l. species in three habitat types in Slovakia between 2011-2013

| Species | | ss | a | g | v | l | s | g+v | a+g | a+g+v | ss+g | ss+g+v+k | ND | TOTAL |
|---|---|---|---|---|---|---|---|---|---|---|---|---|---|---|
| **Bratislava** | | | | | | | | | | | | | | |
| 2011 | Nymphs | 2 | 28 | 13 | 1 | 10 | 7 | | | | | | | **61** |
| | Females | | 38 | 17 | 7 | 3 | 3 | 1 | | | | | | **69** |
| | Males | | 10 | 23 | 2 | 6 | 3 | | | | | | | **44** |
| | Total | 2 | 76 | 53 | 10 | 19 | 13 | 1 | | | | | | **174** |
| 2012 | Nymphs | 1 | 12 | 3 | 7 | | | | | | | | | **23** |
| | Females | | 3 | 2 | 2 | 2 | | | | | | | | **9** |
| | Males | 1 | 3 | 2 | 2 | 1 | | | | | | | | **9** |
| | Total | 2 | 18 | 7 | 11 | 3 | | | | | | | | **41** |
| 2013 | Nymphs | 1 | 4 | | 2 | 1 | | | | | | | | **8** |
| | Females | 3 | 3 | 8 | 12 | 1 | | | | | | | 1* | **28** |
| | Males | 1 | 3 | 5 | 5 | 1 | | | | | | | | **15** |
| | Total | 5 | 10 | 13 | 19 | 3 | | | | | | | 1* | **51** |
| Total | 2011-2013 | 9 | 104 | 73 | 40 | 25 | 13 | 1 | | | | | 1* | **266** |
| **Fúgelka** | | | | | | | | | | | | | | |
| 2011 | Nymphs | 8 | 78 | 65 | 22 | 1 | 1 | 4 | | 1 | 1 | | 3* | **184** |
| | Females | 1 | 6 | 12 | 7 | | | | | | | | | **26** |
| | Males | | 1 | 9 | 2 | | | | | | | | | **12** |
| | Total | 9 | 85 | 86 | 31 | 1 | 1 | 4 | | 1 | 1 | | 3* | **222** |
| 2012 | Nymphs | | 20 | 22 | 7 | 9 | | | | | | | | **58** |
| | Females | | 1 | 12 | 1 | 1 | | | | | | | | **15** |
| | Males | | 1 | 5 | 2 | 2 | | | | | | | | **10** |
| | Total | | 22 | 39 | 10 | 12 | | | | | | | | **83** |
| 2013 | Nymphs | | 10 | 10 | | 1 | | | | | | | | **21** |
| | Females | | 1 | 7 | 2 | | 1 | 1 | | | | | | **12** |
| | Males | | 2 | 5 | 1 | | | | | | | | | **8** |
| | Total | | 13 | 22 | 3 | 1 | 1 | 1 | | | | | | **41** |
| Total | 2011-2013 | 9 | 120 | 147 | 44 | 14 | 2 | 5 | | 1 | 1 | | 3* | **346** |
| **Rozhanovce** | | | | | | | | | | | | | | |
| 2011 | Nymphs | 2 | 25 | 8 | | 1 | | 2 | 1 | | | | 2** | **41** |
| | Females | | 1 | 3 | | 1 | 1 | 1 | | | | | | **7** |
| | Males | | 3 | | | 1 | | | | | | | | **4** |
| | Total | 2 | 29 | 11 | | 3 | 1 | 3 | 1 | | | | **2**** | **52** |
| 2012 | Nymphs | 7 | 19 | 14 | 14 | 2 | | | | | | | | **56** |
| | Females | | 2 | 1 | 2 | 2 | | 1 | | | | 1 | | **9** |
| | Males | 2 | | 2 | 3 | 1 | | 2 | | | | | 1* | **11** |
| | Total | 9 | 21 | 17 | 19 | 5 | | 3 | | | | 1 | 1* | **76** |
| 2013 | Nymphs | 1 | 3 | 9 | 7 | | | | | | | | | **20** |

| | | | | | | | | | | | | | | |
|---|---|---|---|---|---|---|---|---|---|---|---|---|---|---|
| | Females | 1 | | 1 | 1 | | | | | | | | | **3** |
| | Males | | | | 2 | | | | | | | | | **2** |
| | Total | 2 | 3 | 10 | 10 | | | | | | | | | **25** |
| Total | 2011-2013 | 13 | 53 | 38 | 29 | 8 | 1 | 6 | 1 | | | 1 | 1*, 2** | **153** |
| **TOTAL (all sites)** | **2011-2013** | **31** | **277** | **258** | **113** | **47** | **16** | **12** | **1** | **1** | **1** | **1** | **7** | **765** |

*Borrelia burgdorferi* s.l. species. Results of RFLP: ss – sensu stricto, a – *afzelii*, g – *garinii* (+ *bavariensis*), v – *valaisiana*, l – *lusitaniae*, s – *spielmanii*. Results of RLB: g+v – *garinii+valaisiana*, a+g – *afzelii+garinii*, a+g+v – *afzelii+garinii+valaisiana*, ss+g – sensu stricto+*garinii*, ss+g+v+k – sensu stricto+*garinii+valaisiana+kurtenbachii*, * the RFLP profile indicated coinfections, but only *B. garinii* could be identified with RLB, **the RFLP profile indicated coinfections but no samples were left for RLB analysis.
