# Supplementary material for: Spatial and Temporal Variability in Prevalence Rates of Members of the Borrelia burgdorferi Species Complex in Ixodes ricinus Ticks in Urban, Agricultural and Sylvatic Habitats in Slovakia
Source: Microorganisms. 2023 Jun 27;11(7):1666. doi: 10.3390/microorganisms11071666 (PMC10383148; doi:10.3390/microorganisms11071666)
Supplement: Supplementary file 1 [file microorganisms-11-01666-s001.zip › Table S2.pdf]

**Table S2** Prevalence of *Borrelia burgdorferi* s. l. and diversity of *Borrelia* species reported from Slovakia

| Site | Sample | Prevalence (%) | ss | g | v | a | l | s | b | coinf | Method | Ref |
|---|---|---|---|---|---|---|---|---|---|---|---|---|
| Slovakia (W, NS) | A | 3-13 | | | | | | | | | DFM | [33] |
| Bratislava (SW, U) | M | 15.3-21.8 | | | | | | | | | DFM | [34] |
| | F | 14.5-16.9 | | | | | | | | | | |
| | T | 17.8 | | + | | | | | | | | |
| Martinský les (SW, F) | M | 56 | | | | | | | | | IFA, PCR, RFLP | [35] |
| | F | 42 | | | | | | | | | | |
| | T | 49 | | 14 | 14 | 68 | 5 | | | | | |
| Košice (E, U) | N | 9-11 | | | | | | | | | DFM, IB | [36] |
| | A | 15-19 | | | | | | | | | | |
| | T | 2.1-28.4 | + | + | | + | | | | + | | |
| Košice (E, U) | T | 12.8-15.0 | | | | | | | | | DFM | [80] |
| Košice (E, U) | N | 11.1 | | | | | | | | | DFM | [81] |
| | A | 12.7 | | | | | | | | | | |
| Rimavská Sobota (E, U) | N | 14.3 | | | | | | | | | | |
| | A | 27.7 | | | | | | | | | | |
| Michalovce (E, U) | N | 5.2 | | | | | | | | | | |
| | A | 8.3 | | | | | | | | | | |
| Vranov (E, U) | N | 21.4 | | | | | | | | | | |
| | A | 13 | | | | | | | | | | |
| Košice (E, U) | T | 38.3 | | | | | | | | | PCR | [82] |
| Pod. Biskupice (SW, SF) | N | 21.5 | | | 7.1 | 89.3 | 3.6 | | | | PCR, RLB | [20] |
| | A | 31.8 | 1.3 | 27.6 | 10.5 | 52.6 | 2.6 | | | 5.3 | | |
| Malacky (W, UP) | N | 18.7 | | 21.6 | 18.9 | 43.2 | | | | 16.2 | | |
| | A | 40.1 | 1.7 | 20.3 | 20.3 | 49.1 | | | | 8.5 | | |
| Záh. Ves (W, F) | N | 27.8 | 6.7 | 20.0 | 13.3 | 60.0 | | | | | | |
| 1999 | A | 48.5 | 2.1 | 5.2 | 8.3 | 61.4 | 4.2 | | | 18.7 | | |
| 2000 | N | 19.0 | | 6.1 | 45.4 | 48.5 | | | | | | |
| | A | 49.4 | | 7.9 | 50.0 | 38.1 | 3.9 | | | | | |
| Furča (E, F) | N | 1-13 | + | + | | + | | | + *** | | PCR, RFLP | [37] |
| | A | 16-23 | + | + | + | + | | | + *** | | | |
| | T | 13-15 | | | | | | | | | | |
| Rozhanovce (E, F) | N | 8-9 | + | + | | + | | | + *** | | | |

| | | | | | | | | | | | | |
|---|---|---|---|---|---|---|---|---|---|---|---|---|
| | A | 0-5 | + | | | | | | | | | |
| | T | 8 | | | | | | | | | | |
| Malá Ida (E, F) | A | 28 | | + | + | + | | | | | | |
| Slovak Karst (SE, F) | N | 16.9 | + | | | | | | | | PCR, RFLP | [23] |
| | A | 22.5 | | | | | | | | | | |
| | T | 19.3 | 12.7 | 33.3 | 11.1 | 30.2 | 11.1 | | | 1.6 | | |
| C Slovakia | T | 9.4 | + | + | + | + | | | | + | PCR, RLB, RFLP | [83] |
| W Slovakia | T | 5.3 | | + | | + | | | | + | | |
| Martinský les (W, F | T | 32.3 | | 17.7 | 6.5 | 8.1 | | | | | PCR, RLB, RFLP | [84] |
| Krup. planina (SC, F) | T | 28.6 | | 14.3 | 8.6 | 2.9 | | | | 2.9 | | |
| B. Štiavnica (C, A) | T | 36.8 | 5.3 | 15.8 | | 5.3 | | | | 10.6 | | |
| Šúr (SW, PF) | N | 27.8 | | 31.6 | 38.6 | 5.3 | | | | 21.0 | PCR, RLB | [21] |
| | A | 33.6 | | 29.5 | 42.3 | 7.7 | | | | 3.9 | | |
| Malacky (W, U) | N | 14.4 | 3.2 | 3.2 | 12.9 | 77.4 | | | | 3.9 | | |
| | A | 23.2 | 23.1 | 15.4 | 46.2 | 15.4 | | | | | | |
| Drieňovec (SE, F) | N | 14.8 | | | | 50.0 | | | | 50.0 | | |
| | A | 26.7 | | 25.0 | 40.0 | | | | | 10.0 | | |
| Brzotín (SE, F) | N | 20.0 | 7.1 | 21.4 | 7.1 | 57.1 | | | | | | |
| | A | 29.4 | | 40.0 | 30.0 | 20.0 | | | | | | |
| Pod. Biskupice (SW, PF) | T | | 5 | 8 | 3 | 9 | | | | | PCR | [43] |
| E Slovakia 10 sites (mean) | | 10.15 | + | + | + | + | | | | + | PCR, RFLP | [38] |
| Bardejov (NE, U) | | 0-33.3 | | | | | | | | | | |
| Košice (E, U) | | 1.8-18.0 | | | | | | | | | | |
| Martinské hole | N | 16.8 | 3.7 | 6.7 | 3.7 | 20.7 | 62.8 | | | 2.3 | PCR, RFLP | [39] |
| (C, mixed F) | A | 36.2 | | | | | | | | | | |
| | T | 29.2 | | | | | | | | | | |
| Bratislava (SW, U) | N | 7.2 | | | | | | | | | PCR, RFLP | [40] |
| | A | 3.4 | | | | | | | | | | |
| | T | 6.8 | 5.4 | 35.1 | 24.3 | 16.2 | 10.8 | 2.7 | | 5.4 | | |
| Bratislava (SW, U) | N | 20.5 | | | | | | | | | PCR, RFLP | [41] |
| | F | 11.5 | | | | | | | | | | |
| | M | 11.0 | | | | | | | | | | |
| Ž. Studnička (SW, UF) | T | 11.4-13.9 | 5.9 | 23.5 | 14.7 | 42.6 | 7.4 | 5.9 | | | | |
| Horský park (SW, U) | T | 12.0-30.9 | 9.1 | 30.9 | 20.0 | 32.7 | 3.6 | 3.6 | | | | |
| Drieňovec (SE, F) | N | 25.7 | | | | | | | | | PCR, RFLP | [42] |

| | | | | | | | | | | | | |
|---|---|---|---|---|---|---|---|---|---|---|---|---|
| | F | 18.75 | | | | | | | | | | |
| | M | 38.10 | | | | | | | | | | |
| | T | 26.3 | | 16.4 | 14.9 | 61.2 | | | | 6.0 | | |

Explanations

Site description: E – eastern Slovakia, W – western Slovakia, SW – south-western Slovakia, C – central Slovakia, SC – south central Slovakia, E – eastern Slovakia, SE – south-eastern Slovakia, NE – north-eastern Slovakia. NS – not specified, U – urban park, UF – urban forest park, PF – peri-urban forest, F – forest, A – arboretum. Sample: N – nymph, A – adult, M – male, F – female, T – total. *B. burgdorferi* s. l. species: ss – sensu stricto, g – *garinii*, v – *valaisiana*, a – *afzelii*, l – *lusitaniae*, s – *spielmanii*, b – *bavariensis*, + presence (no prevalence is given), *** *B. garinii ospA* serotype 4, coinf – mixed infections. Method: DFM – dark field microscopy, IFA – immunofluorescence, PCR – polymerase chain reaction, RFLP – restriction fragment length polymorphism, IB – immunoblot, RLB – reverse line blot

## References


20. Hanincová, K.; Tarageľová, V.; Koči, J.; Schäfer, S.M.; Hails, R.; Ullmann, A.J.; Piesman, J.; Labuda, M.; Kurtenbach, K. Association of *Borrelia garinii* and *B. valaisiana* with songbirds in Slovakia. *Appl. Environ. Microbiol*. **2003**, *69*, 2825–2830. https://doi.org/10.1128/AEM.69.5.2825-2830.2003

21. Tarageľová, V.; Koči, J.; Hanincová, K.; Kurtenbach, K.; Derdáková, M.; Ogden, N.H.; Literák, I.; Kocianová, E.; Labuda, M. Blackbirds and song thrushes constitute a key reservoir of *Borrelia garinii*, the causative agent of borreliosis in central Europe. *Appl. Environ. Microbiol.* **2008**, *74*, 1289–1293. https://doi.org/10.1128/AEM.01060-07

23. Majláthová, V.; Majláth, I.; Víchová, B.; Derdáková, M.; Peťko, B. *Borrelia lusitaniae* and green lizards (*Lacerta viridis*), Karst Region, Slovakia. *Emerg. Infect. Dis*. **2006**, *12*, 1895–1901. https://doi.org/10.3201/eid1212.060784

33. Kmety, E.; Rehácek, J.; Výrosteková, V. Investigations of ticks for the presence of *Borrelia* in Czechoslovakia. *Zentralbl. Bakteriol. Mikrobiol. Hyg. A.* **1987**, *263*, 468-470. https://doi.org/10.1016/s0176-6724(87)80111-6

34. Drgoňová, M.; Řeháček, J. Prevalence of Lyme borrelia in ticks in Bratislava, Slovak Republic. *Cent. Eur. J. Public Health*. **1995**, *3*, 134-137.

35. Gern, L.; Hu, C.M.; Kocianova, E.; Vyrostekova, V.; Rehacek, J. Genetic diversity of *Borrelia burgdorferi* sensu lato isolates obtained from *Ixodes ricinus* ticks collected in Slovakia. *Eur. J. Epidemiol.* **1999**, *15*, 665-669. https://doi.org/10.1023/a:1007660430664

36. Štepánová-Tresová, G.; Peťko, B.; Štefančíková, A.; Nadzamová, D. Occurrence of *Borrelia burgdorferi* sensu stricto, *Borrelia garini* and *Borrelia afzelii* in the *Ixodes ricinus* ticks from Eastern Slovakia. *Eur. J. Epidemiol.* **2000**, *16*, 105–109. https://doi.org/10.1023/a:1007606623892
37. Lenčáková, D.; Hizo-Teufel, C.; Petko, B.; Schulte-Spechtel, U.; Stanko, M.; Wilske, B.; Fingerle, V. Prevalence of *Borrelia burgdorferi* s.l. OspA types in *Ixodes ricinus* ticks from selected localities in Slovakia and Poland. *Int. J. Med. Microbiol*. **2006**, *296*(Suppl 40), 108-118. https://doi.org/10.1016/j.ijmm.2005.12.012
38. Pangrácová, L.; Derdáková, M.; Pekárik, L.; Hviščová, I.; Víchová, B.; Stanko, M.; Hlavatá, H.; Peťko, B. *Ixodes ricinus* abundance and its infection with the tick-borne pathogens in urban and suburban areas of Eastern Slovakia. *Parasit. Vectors* **2013**, *6*, 238. https://doi.org/10.1186/1756-3305-6-238
39. Rusňáková Tarageľová, V.; Mahríková, L.; Selyemová, D.; Václav, R.; Derdáková, M. Natural foci of *Borrelia lusitaniae* in a mountain region of Central Europe. *Ticks Tick Borne Dis*. **2016**, *7*, 350-356. doi: 10.1016/j.ttbdis.2015.12.006
40. Chvostáč, M.; Špitalská, E.; Václav, R.; Vaculová, T.; Minichová, L.; Derdáková, M. Seasonal patterns in the prevalence and diversity of tick-borne *Borrelia burgdorferi* sensu lato, *Anaplasma phagocytophilum* and *Rickettsia* spp. in an urban temperate forest in south western Slovakia. *Int. J. Environ. Res. Publ. Health* **2018**, *15*, 994. https://doi.org/10.3390/ijerph15050994
41. Vaculová, T.; Derdáková, M.; Špitalská, E.; Václav, R.; Chvostáč, M.; Rusňáková Tarageľová, V. Simultaneous occurrence of *Borrelia miyamotoi*, *Borrelia burgdorferi* sensu lato, *Anaplasma phagocytophilum* and *Rickettsia helvetica* in *Ixodes ricinus* ticks in urban foci in Bratislava, Slovakia. *Acta Parasitol.* **2019**, *64*, 19-30. https://doi.org/10.2478/s11686-018-00004-w
42. Mtierová, Z.; Derdáková, M.; Chvostáč, M.; Didyk, Y.M.; Mangová, B.; Rusňáková Tarageľová, V.; Selyemová, D.; Šujanová, A.; Václav, R. Local population structure and seasonal variability of *Borrelia garinii* genotypes in *Ixodes ricinus* ticks, Slovakia. *Int. J. Environ. Res. Publ. Health*. **2020**, *17*, 3607. https://doi.org/10.3390/ijerph17103607
43. Subramanian, G.; Sekeyova, Z.; Raoult, D.; Mediannikov, O. Multiple tick-associated bacteria in Ixodes ricinus from Slovakia. *Ticks Tick Borne Dis*. **2012**, *3*, 406-410. https://doi.org/10.1016/j.ttbdis.2012.10.001
80. Nadzamová, D.; Petko, B.; Stefancíková, A.; Cisláková, L.; Stěpánová, G.; Seba, M. Potential foci of Lyme borreliosis in towns. *Cent. Eur. J. Publ. Health* **2000**, *8*, 176-178.

81. Peťko, B.; Štefančíková, A.; Nadzamová, D.; Stanko, M.; Siuda, K.; Karbowiak, G.; Winiarczyjk, S. Epizootiological aspects of Lyme borreliosis in the city agglomerations of the Carpathian regions of Slovakia and Poland and their peripheral parts. In *Stawonogi Pasożyty i nosiciele*; Buczek A., Błaszak C., Eds.; Wyd. KGM: Lublin, Poland, 2001; pp. 157-164.
82. Derdakova, M.; Halanova, M.; Stanko, M.; Stefancikova, A.; Cislakova, L.; Pet'ko, B. Molecular evidence for *Anaplasma phagocytophilum* and *Borrelia burgdorferi* sensu lato in *Ixodes ricinus* ticks from eastern Slovakia. *Ann. Agric. Environ. Med.* **2003**, *10*, 269-271.
83. Smetanová, K.; Schwarzová, K.; Kocianová, E. Detection of *Anaplasma phagocytophilum*, *Coxiella burnetti*, *Rickettsia* spp., and *Borrelia burgdorferi* s.l. in ticks and wild living animals in western and middle Slovakia. *Ann. N. Y. Acad. Sci.* **2006**, *1078*, 312–315. https://doi.org/10.1196/annals.1374.058
84. Smetanová, K.; Burri, C.; Pérez, D.; Gern, L.; Kocianová, E. Detection and identification of *Borrelia burgdorferi* sensu lato genospecies in ticks from three different regions in Slovakia. *Wien. Klin. Wochenschr*. **2007**, *119*, 534–537. https://doi.org/10.1007/s00508-007-0851-y
